# A Remarkable Case of Petechiæ, Unaccompanied by Fever

**Published:** 1799-07

**Authors:** J. F. Davis

**Affiliations:** Bath.


### *A remarkable Caſe of Petechiæ, unaccompanied by Fever.*

[*Communicated by* J. F. DAVIS, *M.D. of Bath.*]

THE publication of extraordinary caſes is not, perhaps, of ſo much utility as is generally imagined; for ſince they rarely occur, the information communicated cannot be of extenſive advantage. A ſucceſsful mode of treating one common diſeaſe, exceeds in value the detail of many curious and uncommon complaints. It belongs to ſcience, however, to amuſe, as well as to inſtruct mankind. With this view I have drawn up the following caſe, which, though not entirely new*, contains ſome ſtriking peculiarities:—

Monday, March 11th, I was deſired by Mr. GOLDSTONE, ſurgeon, in this city, to viſit a boy labouring under a peculiar diſeaſe, which he conſidered as partaking very much of the nature of ſcurvy. The whole ſurface of his body was covered with light, red, purple, and blackiſh ſpots, from the ſmalleſt ſize poſſible to that of a pea. They did not appear to be thicker on one part than on another. The arms were covered with them, like the body, but there were not many on the legs; on theſe, however, and on one of the arms, were larger ſpots, of a livid hue, commonly known by the name of vibices. That on the hand, his parents attributed to a bruiſe; which he had received before his illneſs; but as they could not account for thoſe on the legs, it ſeems probable that they aroſe from a different cauſe. His gums bled profuſely, and were in a very putrid ſtate. Reſpiration was quick and laborious, aud his breath extremely offenſive; urine natural; bowels coſtive, there having been no ſtool for two days; he was ſenſible, but could not ſpeak. It was evident that he could not live long; he died in the evening. I learned from his friends, that the eruption appeared firſt on Sunday, 13th of February, but that he continued to follow his uſual occupation till the 3d of March,

* See Withers, on the Asthma, p. 422.—Duncan's Cases and Observations, p. 90, Memoirs of the Medical Society of London, Vol. IV.—Monthly Review, for 1796, Vol. XIX. p. 478.

March, complaining only of being weaker than formerly. The ſpots had during this period increaſed in ſize. He was then ſeized with ſhivering, which was not followed by any perceptible fever. After that he coughed frequently, and blood was diſcharged from the lungs. On Friday, March 8th, he vomited near a quart of grumous blood, and his ſtomach was thereby much relieved from an oppreſſion which had been extremely painful to him. Afterwards he diſcharged from his ſtomach, at different times, lumps of the ſame kind, but in ſmaller quantity.

In my endeavours to diſcover the cauſe of this ſtrange appearance, great difficulties have occurred. The boy had not lived on food which was likely to occaſion ſuch a diſeaſe. He ſerved a confectioner, and was fed with a variety of freſh and wholeſome food. He ſlept at home, and though the houſe (his parents being poor) was ſmall and dirty, he was not more expoſed to danger on this account than his brothers, who bore on their cheeks the ruddy hue of health. I found that he had been much employed in the ice-houſe, and had frequently complained of the intenſe cold to which he was expoſed in that ſituation. Could cold, together with damp and foul air, which abounds in thoſe places, have produced the diſeaſe under conſideration? Writers on ſcurvy admit that cold has conſiderable influence in the production of that diſeaſe, for, *cæteris paribus*, it is much more common in cold than in warm climates. Cold moreover occaſions an appearance on the hands and faces of children who have been long expoſed to it, that it denotes a ſtate of the capillaries approaching to that which takes place in petechiæ. No one, I believe, will deny that pure air is neceſſary to a healthy ſtate of the blood.

Petechiæ, as a ſymptom of fever, is well known; but as an idiopathic diſeaſe, proving fatal*, they are, I believe, extremely rare—ſo rare, indeed, that Dr. Cullen has the following remark:—"Cum in quibuſdam febribus, vel intermittentibus, vel continuis, in quibuſvis etiam exanthematis et profluviis, modo in iis morbis ſit quædam ad putredinem proclivitas, appareant petechiæ; has pro efflorescentia ſymptomatica, potius quam pro exanthemate idiopathico habere voluit†." In this caſe they were certainly not ſymptomatic of fever. It bears a very near relation to ſcurvy, but ſome circumſtances prove the difference.

To

* In the cases to which I have before alluded, a cure was effected by means of bark, wine, and acids. The ſubject of my case did not seek relief till it was too late. The most judicious plan of treatment was nevertheless employed by the surgeon who attended.

† Synop. Nos. Method. p. 152.

