# Errata in Vol. I

**Published:** 1799-08

**Authors:** 


No. v. p 421, l. 22, dele the word "*it,*" before denotes.

No. v. p. 421, l. 25, for "*is* well known," read, "*are* well known."

